# Incretin‐Based Therapies in Alzheimer's and Parkinson's Disease: Advancing Neuroprotection With Dual and Triple Agonists—A Review

**DOI:** 10.1002/hsr2.71065

**Published:** 2025-07-15

**Authors:** Ousman Mohammed, Tsehayneh Kelemu

**Affiliations:** ^1^ Department of Medical Biochemistry, School of Medicine, College of Health Sciences Addis Ababa University Addis Ababa Ethiopia; ^2^ Department of Medical Laboratory Sciences, College of Medicine and Health Sciences Wollo University Dessie Ethiopia

**Keywords:** Alzheimer disease, glucagon‐like peptide1 receptors, glucose‐dependent insolinotropic polypeptide, incretin signaling, insulin signaling, Parkinson disease, therapeutic targets

## Abstract

**Background:**

Alzheimer's disease (AD) and Parkinson's disease (PD) are progressive neurodegenerative disorders with significant cognitive and motor impairments, affecting millions globally. Current treatments offer limited efficacy, prompting the exploration of new therapeutic approaches.

**Aim:**

To discuss the intricate relationship between incretin and insulin signaling pathways and their relevance to the pathogenesis and treatment of Alzheimer's and Parkinson's diseases.

**Methods:**

A comprehensive literature review was conducted using a variety of search engines, including Google Scholar, PubMed Central, Scopus, Web of Science, and others.

**Results:**

Emerging evidence highlights disrupted insulin signaling in AD and, to a lesser extent, in PD, suggesting that insulin plays a key neuroprotective role. Incretins, such as GLP‐1 and GIP, which enhance insulin signaling, have shown potential in preclinical and clinical studies. Incretin‐based therapies, particularly GLP‐1/GIP receptor agonists, have demonstrated promising effects by addressing several pathological processes, including oxidative stress, inflammation, misfolded protein aggregation, and insulin resistance. Dual agonists like DA‐CH3, DA5‐CH, and DA4‐JC have proven superior in crossing the blood‐brain barrier and offering improved neuroprotection in comparison with conventional GLP‐1 agonists. Triple agonists provide even greater neuroprotective benefits, highlighting their potential as disease‐modifying therapies for AD and PD.

**Conclusion:**

While GLP‐1 and GIP analogs hold promise in modulating early neurodegenerative processes, their efficacy likely depends on timely intervention before permanent neuronal damage occurs.

## 1 Introduction

Neurodegenerative diseases, such as Alzheimer's and Parkinson's, are progressive neuronal dystrophic conditions causing cognitive decline, motor impairments, and significant morbidity worldwide [1, 2, 3, 4]. AD is responsible for roughly 60%–70% of dementia cases internationally, with a global prevalence of around 50 million cases [5]. Increases in population ageing and growth have led to estimates that cases will triple by 2050, representing a huge economic burden and an increasing challenge to patients, carers, and healthcare systems [6]. On the other hand, there were 6.1 million PD sufferers globally, though with substantial geographical variations and a higher prevalence in men than women [7]. It was estimated that there would be a doubling of cases in the next 30 years [8].

Targeted interventions are necessary because current therapies target misfolded proteins like tau, α‐synuclein, and Aβ42. However, there is mounting evidence that early brain insulin signaling dysfunction is linked to the pathogenesis of AD and PD [9, 10, 11]. Targeting brain insulin resistance and incretin signaling, both strongly associated with cognitive decline, has gained attention as a result of the lack of effective treatments for AD and PD despite extensive research. Previously recognized for their metabolic functions, incretins such as GLP‐1 and GIP are now understood to have neuroprotective and regenerative properties, making them promising therapeutic targets in neurodegenerative diseases [12, 13, 14].

## 2 The Link Between Insulin Signaling and Alzheimer's and Parkinson's Disease

Insulin signaling is crucial for brain function, regulating glucose metabolism, protecting neurons, maintaining synaptic integrity, and supporting cognitive processes. It originates from pancreatic β‐cells and local production in neurons [15, 16]. The brain should now be included on the list of organs impacted by insulin resistance and illnesses caused by changes in glucose metabolism that affect neurons and glial cells [17, 18, 19]. People with diabetes are at risk of getting Alzheimer's disease, as clearly proven by the Rotterdam research in the late 1990s (and many other articles thereafter) [20]. These two metabolic illnesses are also very closely related from a pathophysiological standpoint, not just because of insulin resistance but also because of profound disruptions in glucose metabolism that damage neurons and glial cells. The brain (2% of organ weight) requires 25% of total energy per day, primarily sugar, for optimal ATP generation [21]. One of the most critical jobs of glial cells is to produce lactate from glycolysis and shuttle it into neurons to power the Krebs cycle; however, due to insulin resistance, this is not operating properly, resulting in neuron malfunction. These strong ties between AD and diabetes explain why AD is commonly referred to as type 3 diabetes [22]. Aside from their own neuroprotective effects, one major role of GLP‐1/GIP analogs is to overlap insulin resistance and restore insulin sensitivity (not only in the brain, but in many other insulin‐sensitive organs [23, 24]. However, disruption can lead to protein aggregation, neuroinflammation, and mitochondrial dysfunction. Studies show significant insulin resistance in AD brains [25, 26]. For example, phosphatidylinositol 3‐kinase (PI3K) signaling dysfunction activates glycogen synthase kinase 3 beta (GSK‐3β), leading to hyperphosphorylation of tau protein, a major component of neurofibrillary tangles [17, 27]. Intranasal insulin treatment significantly improved working memory and reduced anxiety‐like behavior in an amyloid‐β‐induced AD rat model. This effect is associated with increased levels of brain‐derived neurotrophic factor (BDNF) and its receptor tropomyosin‐related kinase B (TrkB) in the hippocampus [28]. Insulin resistance in AD drives cognitive loss and altered IRR‐1 phosphorylation in neurons, suggesting potential involvement in neurodegenerative processes [17, 29, 30].

Insulin resistance can lower the levels of insulin‐degrading enzyme (IDE), a key regulator of Aβ levels in neurons and microglia [31, 32]. By cleaving Aβ at several locations, IDE lessens the neurotoxicity and buildup of misfolded proteins. Elevated IDE expression in cultured astrocytes promotes Aβ plaque breakdown via extracellular signal‐regulated kinase (ERK) signaling [33]. Insulin resistance in the brain may affect PD clinical features by decreasing IDE expression, increasing GSK3β activity, and causing tau hyperphosphorylation. This resistance may also restrict Aβ elimination, leading to toxic plaque development and tau pathology [34, 35].

## 3 Intranasal Insulin Administration

Intranasal delivery offers a noninvasive solution for treating brain disorders, bypassing the neurovascular barrier and avoiding systemic effects like hypoglycemia, making it a more realistic treatment option [36, 37]. Insulin improves Aβ42 elimination, inhibits tau hyperphosphorylation, and improves synaptic plasticity signaling pathways. Male Wistar rats given 2 IU of insulin intranasally saw rapid brain distribution and insulin signaling pathway activation. Secondary activation of AMPK and CaMK, as well as increases in insulin and C‐peptide levels in the brain and plasma, suggested region‐specific feedback reactions [38]. A series of clinical trials were performed to explore the notion that regulating the brain insulin pathway has therapeutic effects in AD patients [39, 40].

Studies have demonstrated improved memory, attention, and thinking skills in individuals with AD or mild cognitive impairment (MCI), particularly in those who do not carry the APOEε4 gene [41, 42]. However, among APOEε4 carriers who show lower therapeutic response and altered insulin signaling [43, 44]. Long‐term use raises concerns about insulin resistance, even if it could be essential to notice advantages in APOEε4‐positive patients [45, 46]. Alternative approaches, including GLP‐1 receptor agonists, might provide more reliable neuroprotective effects in light of these drawbacks [13].

## 4 Glucagon‐Like Peptide‐1 and Glucose‐Dependent Insulinotropic Polypeptide

As insulin appears to be derived via insulin receptor‐dependent signaling, therapy might not work in later stages of AD due to a considerable decrease in cell surface insulin receptors [47]. As an alternative to repeatedly using insulin in the initial phases of the illness, incretin analogs activate insulin‐signaling pathways and do exist and may be beneficial therapeutically in late AD. GLP‐1 and GIP analogs have changed how we manage type 2 diabetes. They not only help control blood sugar but also offer heart, kidney, and liver protection. This broad range of benefits shows they might also be useful for brain issues related to metabolism [48]. GLP‐1 increases phosphorylated IRS‐1 and Akt, circumventing insulin receptors and enhancing insulin‐related signaling pathways [49]. Most of the insulin synthesis is driven by the two incretins, GIP and GLP‐1. GLP‐1, a 30‐amino acid peptide, is largely produced by intestinal L cells, whereas GIP, a 42‐amino acid peptide, is produced by K cells in response to nutritional intake [50]. Activating GLP‐1 receptors (GLP‐1Rs) in pancreatic islet cells can stimulate insulin secretion, increase β‐cell proliferation, reduce β‐cell apoptosis, and inhibit glucagon secretion in T2DM patients [51].

GLP‐1Rs are expressed in different tissues, including the brain, indicating that they could be used in disorders other than T2DM [52, 53]. Interestingly, GLP‐1 is expressed in neurons and functions as a neurotransmitter. GLP‐1's activities are through the mediation of the GLP‐1R, a seven‐transmembrane‐spanning G protein‐coupled receptor (GPCR). It stimulates a GPCR subunit, which activates adenyl cyclase and increases cAMP synthesis [54]. Cyclic AMP activates PKA, a critical component that phosphorylates and activates various downstream effectors that regulate protein synthesis and antiapoptotic mechanisms. GLP‐1 works primarily through the PI3‐K and MAPK pathways [55]. However, both incretins are rapidly inactivated by the enzyme dipeptidyl peptidase IV (DPP‐IV), resulting in short half‐lives and preventing their clinical use [51].

Dipeptidyl peptidase‐IV (DPP‐IV) cleaves peptides at the N‐terminal proline or alanine, but it can also act more slowly on serine, glycine, and valine [51, 56]. To improve GLP‐1 stability, analogs such as exendin‐4 replace alanine with glycine, which increases their half‐life and resistance to DPP‐IV degradation [57]. The first GLP‐1R agonist authorized for type 2 diabetes was exenatide, which was followed by liraglutide and semaglutide [58]. In addition to controlling blood sugar, GLP‐1R agonists have neuroprotective effects by encouraging the growth of new neurons and lowering oxidative stress, neuroinflammation, and apoptosis [57].

**Figure 1:**
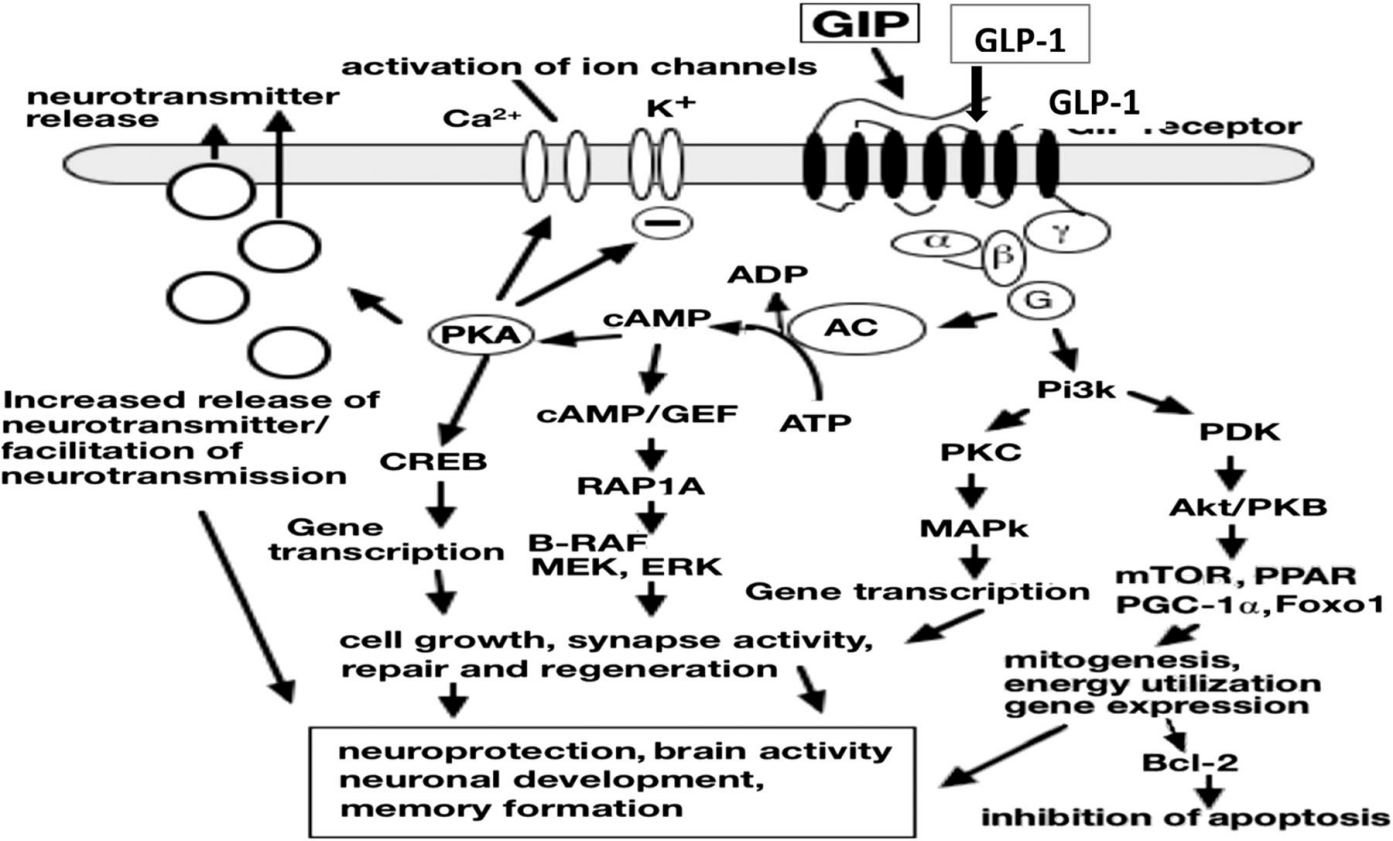
The GIP functions within the brain. The GIP receptor triggers the adenylyl cyclase (AC), increasing intracellular cAMP levels and activating PKA. This can boost vesicle release, glucose‐stimulated insulin production, and synaptic neurotransmitter release. AC also produces ADP, which affects ATP‐sensitive K+ channels. This leads to increased voltage‐dependent l‐type Ca2+ channels and cytosolic Ca2+ levels, facilitating GIP's rapid influence on synaptic transmission. PI3K levels increase G‐protein‐dependent, activating MAPKs, Akt/PKB, mTOR, PPARα/δ/γ, promoting cell growth, reducing inflammation, initiating mitogenesis, and inhibiting apoptosis via Bcl‐2 and other pathways [63]. Abbreviations: ADP = adenosine diphosphate, Akt/PKB = protein kinase B complex, Bcl‐2 = B‐cell lymphoma 2, cAMP–GEFs = cAMP–guanine‐nucleotide‐exchange factors, ERK = extracellular signal‐regulated kinase, MEK = MAPK kinase, MAPK = mitogen‐activated protein kinase, mTOR= mammalian target of rapamycin, PDK=phosphatidylinositol dependent kinase, PGC‐1α = Peroxisome proliferator‐activated receptor gamma coactivator 1‐alpha, Pi3K = phosphatidylinositol 3 kinase, PKA = protein kinase A, PPAR = peroxisome proliferator‐activated receptor, RAP1A = Ras‐related protein Rap‐1A, Ras = rat sarcoma virus peptide.

Neuroprotective mechanism of GIP/GLP‐1: The activation of GIP and GLP‐1 receptors, which are widely expressed in neurons, astrocytes, and microglia, has anti‐inflammatory, neuroprotective, and insulin‐sensitizing effects [59, 60]. The cAMP/PKA‐CREB and PI3K‐MAPK pathways are activated by GLP‐1/GIP upon receptor binding, which also activates the Gαs and Gβγ subunits [61, 62]. To support brain repair and cognitive function, these cascades improve synaptic plasticity, neurotransmitter release, neuronal survival, and antiapoptotic and anti‐inflammatory responses (Figure 1) [63, 64].

## 5 The Role of GIP Analogs as Neuroprotectives in the AD Model

There is a developing study on the potential role of GIP agonists in AD treatment. While GIP is most generally linked to metabolic management, it also has effects on the central nervous system, including neuroprotective and anti‐inflammatory properties [14, 49]. Activation of GIP receptors in the brain has been shown to have potential benefits in animal models of neurodegenerative diseases, including AD [65, 66]. APP/PS1 transgenic mice treated with daily intraperitoneal d‐Ala²GIP for 8 weeks showed significant reductions in amyloid plaque burden, cortical synaptic loss, hippocampal LTP to wild‐type levels, and memory preservation [9, 67]. The medication significantly reduced chronic inflammation, oxidative stress, DNA damage, and astrocyte activation. Memory formation and recall impairments are also reversed, and synaptic plasticity in the hippocampus is normalized [11, 67]. According to another study, GLP‐1/GIP treatment improved β‐amyloid clearance, decreased mitophagy and neuroinflammation, normalized autophagy, and restored mitochondrial function and insulin signaling [11, 24, 63].

## 6 Neuroprotective Effects of GIP Analogs in the PD Model

d‐Ala2‐GIP‐glu‐PAL, an analog, had good neuroprotective benefits against toxicity caused by 1‐methyl‐4‐phenyl‐1, 2, 3, and 6‐tetrahydropyridine (MPTP). d‐Ala2‐GIP‐glu‐PAL improved motor function, restored tyrosine hydroxylase expression, minimized inflammation, and normalized cAMP/PKA/CREB second messenger signaling in the brain [24, 48, 68]. Interestingly, d‐Ala2‐GIP‐glu‐PAL also improved synapses and BDNF while decreasing oxidative stress and lipid peroxidation [69]. Moreover, d‐Ala2‐GIPglu‐PAL treatment decreased α‐synuclein accumulation [70]. Another study revealed that d‐Ala2‐GIP‐glu‐PAL treatment improved motor function and greatly increased the number of dopamine‐producing neurons that expressed the enzyme tyrosine hydroxylase [69]. Loss of BDNF, as seen in the PD model, results in loss of synaptic function and impaired dopaminergic transmission [71].

Furthermore, GIP therapy demonstrated neuroprotective effects in a moderate‐traumatic brain injury paradigm in rats [72]. Similarly, d‐Ala2‐GIP treatment protects dopaminergic neuronal activity and synthesis, reverses insulin desensitization, normalizes mitochondrial activity and mitogenesis, and reduces mitophagy [73]. The d‐Ala2‐GIP therapy significantly decreased malondialdehyde levels and increased glutathione synthesis. MPTP treatment reduced striatal dopamine and its metabolites, homovanillic acid and 3, 4‐dihydroxyphenylacetic acid (DOPAC), but d‐Ala2‐GIP treatment restored striatal dopamine levels [74]. In another PD model, treatment with GIP continuously delivered by an osmotic minipump prevented 6‐hydroxy‐dopamine (6‐OHDA) toxicity and decreased spinning movements in the lesioned rats [75].

## 7 Glucagon‐Like Peptide‐1 Analogs in AD

GLP‐1 receptor agonists alleviate AD symptoms by promoting AMPK signaling, which minimizes amyloid‐beta production and neuroinflammation while enhancing plaque clearance. GLP‐1 receptor agonists (e.g., exenatide, liraglutide, lixisenatide, semaglutide, dulaglutide) improve insulin signaling, reduce Aβ and tau pathology, modulate MAPKs and GSK‐3β, decrease neuroinflammation, and restore cognition in Alzheimer's disease animal models, showing strong neuroprotective potential [76, 77, 78].

**Preclinical evidence:** Preclinical data indicate that GLP‐1 receptor agonists hold great promise for preventing the progression of dementia. Exendin‐4 reduced AD hallmarks, including amyloid‐β deposition, aberrant glycoprotein glycan expression, cognitive and memory deficits, IRS‐1 serine phosphorylation, and higher blood glucose levels [79]. In an AD animal model, exendin‐4 treatment activates GLP‐1R, decreases mitochondrial toxicity, improves recognition and memory impairment, reduces neuroinflammatory cytokines, decreases amyloid‐β‐induced oxidative stress, and increases levels of synaptic proteins [80, 81, 82, 83, 84].

Dulaglutide similarly showed efficacy against AD tauopathy, reducing tau hyperphosphorylation in streptozotocin‐treated mice via improved PI3K/AKT/GSK3β signaling [85]. Semaglutide was demonstrated to provide protection against Aβ25–35 in SH‐SY5Y cell cultures by promoting autophagy and preventing apoptosis [86]. A systematic review study including 17 studies demonstrated that semaglutide has significant neuroprotective effects in animal models, improving cognitive function, reducing neuronal damage, and showing therapeutic promise for conditions like stroke, obesity‐related cognitive impairment, and sepsis‐induced brain dysfunction [87]. Furthermore, The EVOKE and EVOKE+ trials are pivotal phase 3 studies evaluating the potential of semaglutide, a GLP‐1 receptor agonist, as a disease‐modifying therapy for early‐stage symptomatic AD. These are the first large‐scale trials on semaglutide's efficacy, safety, and tolerability [88].

Liraglutide's neuroprotective benefits were accompanied by normalization of brain GLP‐1 signaling and PKA levels [42, 89]. In astrocytes treated with Aβ, GLP‐1 increased BDNF secretion, restored astrocyte function, and reversed neuronal loss via cAMP/PKA activation. It is worth noting that the number of synapses increased even at this late stage of AD, implying that synaptogenesis had occurred, which is part of the GLP‐1 growth factor's physiological effects [10, 89]. Treatment with liraglutide improved spatial memory in 5xFAD animals; this effect was linked to increased aerobic glycolysis in astrocytes, which strengthened cell support and promoted neuronal survival [90].

Moreover, it was found that liraglutide increased the levels of IDE and phosphorylated insulin receptors, indicating that the neuroprotective effects of this GLP‐1 receptor agonist may be partially mediated by the restoration of cerebral insulin signaling [91]. As the disease progresses in AD patients, there is a correlation between decreased energy metabolism and decreased insulin signaling, which is reflected in the reduced uptake of glucose [92]. However, one study indicated that liraglutide therapy did not significantly reduce amyloid‐β plaque load in transgenic APP/PS1 animal models [93].

Another agonist of the GLP‐1 receptor, lixisenatide injection, significantly reduced both neurofibrillary tangles and amyloid plaques within the hippocampi in 12‐month‐old APP/PS1/tau transgenic mice [94]. Semaglutide inhibits apoptosis and promotes autophagy in SH‐SY5Y cells, making it effective in treating β‐amyloid disease [86, 95]. CJC‐1131, a new GLP‐1 receptor agonist with an extended half‐life, was found to enhance LTP, restore PKA levels, and protect against cognitive decline [96]. Preclinical models demonstrate how GLP‐1 receptor agonists can prevent amyloid plaque formation, reduce inflammation, improve cognition, and promote neurogenesis [83, 85, 86].

**Clinical trials:** Clinical trials have investigated the ability of GLP‐1R agonists to treat AD. In AD patients, liraglutide therapy for 6 months prevented a reduction in cerebral glucose metabolism but had no impact on cognitive scores or amyloid load, according to a pilot trial that included 18 patients receiving active treatment or placebo (*n* = 20) [97]. During a phase II clinical study, liraglutide greatly improved glucose transport at the BBB, increasing its metabolic rate and reversing glucose transport defects in the brain that are frequently linked to AD pathology [98]. Without improving cognitive function, liraglutide boosted brain connectivity [99], but a phase II study revealed that it enhanced cognition and AD biomarkers and was well tolerated [100].

In an 18‐month randomized pilot study assessing exenatide's impact on AD, it was reported that 11 patients receiving twice‐daily exenatide medication had lower levels of Aβ42 in extracellular vesicles at 18 months compared to 10 individuals receiving a placebo. Nonetheless, patients treated with exenatide or a placebo had comparable neuropsychological and MRI results [101]. GLP‐1R stimulation, using exendin‐4 therapy, effectively reduced amyloid‐β‐induced microglial activation, neuroinflammation, and neuronal survival by lowering TNF‐α, C1q, and IL‐1α inducers [14, 102]. In addition, sitagliptin (SITG) shows neuroprotective effects by inhibiting key enzymes involved in AD, including AChE, BACE‐1, DPP‐4, and GSK‐3β. It reduces neuroinflammation and oxidative stress while regulating apoptotic markers [3, 4]. Likewise, a systematic review study found GLP‐1 receptor agonist therapy may offer potential metabolic and neuroprotective benefits [103].

Comparing preclinical and clinical data on antidiabetic medicines for AD reveals significant limitations. Most preclinical studies use transgenic mouse models based on familial AD mutations, while over 99% of AD patients have the sporadic form [104]. This discrepancy raises questions about the translational relevance of animal‐to‐human trials, particularly regarding the onset and progression of insulin action in the brain [10]. Despite these challenges, preliminary clinical studies suggest that GLP‐1 receptor agonists show promise as a neuroprotective treatment for AD patients and those at risk (Table 1).

**Table 1:** Summary table comparing the main preclinical and clinical studies on GLP‐1 receptor agonists in Alzheimer's disease.

Model/Design	GLP‐1R Agonist	Findings	Mechanistic Insight
AD mouse model [79]	Exendin‐4	Reduced Aβ and cognitive deficits	Insulin signaling improvement
AD model + neurons [82, 83]	Exendin‐4	Restored memory and synaptic proteins	Anti‐inflammatory and antioxidant effects
STZ‐induced mice [85]	Dulaglutide	Reduced tau pathology	PI3K/AKT/GSK3β modulation
SH‐SY5Y cells [86]	Semaglutide	Reduced apoptosis	Enhanced autophagy
Systematic review [87]	Semaglutide	Improved cognition	Broad neuroprotection
APP/PS1 and hTau mice [93]	Liraglutide	Reduced tau, improved survival	Anti‐inflammatory, tau‐targeting
Astrocytes and 5xFAD mice [89, 90]	Liraglutide	Enhanced synaptic markers	Metabolic‐neuroprotective link
APP/PS1 mice [10]	Liraglutide	Restored insulin signaling	Insulin sensitization
APP/PS1/tau mice [94]	Lixisenatide	Reduced Aβ and tau	Dual pathology targeting
AD model [96]	CJC‐1131	Improved cognition	PKA pathway restoration
Phase I/II trials [97, 98]	Liraglutide	Preserved brain metabolism	Early biomarker effects
Phase II (*n* = 200) [100]	Liraglutide	Improved brain volume, function	Clinical promise
Pilot RCT [101]	Exenatide	Lowered Aβ42 in vesicles	Biomarker impact
Preclinical studies [14, 102]	Exendin‐4	Reduced inflammation	Anti‐microglial action

## 8 Glucagon‐Like Peptide‐1 Receptor Analogs in PD

Current PD treatments, such as deep brain stimulation and dopamine replacement therapy, primarily address symptoms without slowing disease progression. Long‐term use of levodopa, the cornerstone of PD treatment, can lead to side effects like dyskinesia and fails to prevent disease advancement [105]. Thus, therapies that target underlying disease mechanisms are urgently needed [106, 107]. Growing evidence suggests that incretin‐based therapies offer neuroprotective potential in PD by reducing stress, inflammation, improve insulin resistance, stabilize mitochondrial function, improve cognitive and motor function and promoting neuronal survival [108, 109]. These therapies have been shown to improve motor and cognitive deficits in animal models [110]. GLP‐1 activates the PI3K/Akt and MAPK/ERK pathways, inhibiting α‐synuclein accumulation, microglial activation, and apoptosis, while enhancing synaptic plasticity and maintaining the blood‐brain barrier [111, 112, 113]. Preclinical studies show that exendin‐4, a GLP‐1 agonist, promotes neurite outgrowth, decreases misfolded protein accumulation, and protects dopaminergic neurons by inhibiting microglial activation [114]. Extended‐release exendin‐4 (PT302) has shown potential in reducing motor deficits and neurodegeneration in PD models [110].

In MPTP‐induced PD models, liraglutide and semaglutide effectively reduced α‐synuclein burden and inflammation while improving motor impairments and raising tyrosine hydroxylase levels, with semaglutide showing greater neuroprotective effects than liraglutide [114]. Additionally, the long‐acting agonist for the GLP‐1 receptor, such as NLY01, prevented dopaminergic loss and behavioral abnormalities and inhibited astrocyte conversion to the toxic A1 phenotype, a key driver of PD inflammation [115].

**Clinical evidence:** A Phase II trial enrolled 57 participants to explore the safety and efficacy of once‐daily liraglutide treatment for PD. After 54 weeks of therapy, the non‐motor symptom scores (NMSS) in the medication group improved by 6.6 points, whereas the NMSS in the placebo group worsened by 6.5 points. This resulted in an adjusted mean difference of 13.1 points (*p* = 0.07). The study found that liraglutide significantly improved the daily lives of PD patients beyond the benefits of l‐Dopa in everyday activities [109].

Exenatide treatment for 12 weeks improved mood and reduced depression markers in moderate‐stage PD patients, with effects lasting up to 48 weeks [116]. Studies suggest exenatide interacts with PD through the Akt, mTOR, and insulin signaling pathways [117, 118]. Post‐hoc analysis showed better responses in patients with milder disease, and those with obesity or insulin resistance had higher baseline cognitive scores [118]. Exenatide increased IRS‐1 phosphorylation and improved Akt and mTOR expression at 48 and 60 weeks, highlighting the role of PI3K‐Akt signaling in its neuroprotective effects [119, 120]. Restoring Akt and mTOR signaling may lower inflammation, promote cell survival, and protect dopaminergic neurons [10, 64].

A phase II trial with 62 idiopathic PD patients found that exenatide treatment over 48 weeks significantly improved motor function during the off‐medication period compared to placebo [121]. Younger patients with milder disease responded better, and exenatide also improved cognitive outcomes in patients with obesity or insulin resistance, suggesting a potential role for GLP‐1 in brain insulin signaling [119]. Ongoing phase III trials (NCT04232969) are investigating exendin‐4 in PD patients [122]. In mouse models, semaglutide has shown promising effects in reducing motor impairments, α‐synuclein accumulation, and inflammation, while protecting dopaminergic neurons [114]. Furthermore, a recent phase 2 clinical trial (LIXIPARK; NCT03439943) included 156 participants randomly assigned to lixisenatide or placebo groups assessed the neuroprotective effects of lixisenatide in patients with early PD. After 12 months, motor symptoms assessed by MDS‐UPDRS part III remained stable in the lixisenatide group (mean change −0.04), while the placebo group experienced a deterioration (mean change +3.04), resulting in a significant between‐group difference of 3.08 points (95% CI, 0.86–5.30; *p* = 0.007). After a 2‐month washout, motor scores continued to favor lixisenatide. Although secondary outcomes indicated no significant differences, lixisenatide was associated with higher gastrointestinal side effects, including nausea (46%) and vomiting (13%) [123].

## 9 Glucagon‐Like Peptide‐1 (GLP‐1)‐Glucose‐Dependent Insulinotropic Polypeptide (GIP) Receptor Co‐Agonists for AD and PD Treatment

By lowering inflammation, improving synaptic and mitochondrial function, and increasing brain penetration, dual GIP/GLP‐1 receptor agonists such as DA3‐CH, DA4‐JC, and DA5‐CH demonstrated better neuroprotective effects than liraglutide in AD and PD models (Table 2) [63, 124, 125]. Tirzepatide (50 and 100 nmol/kg, sc) enhanced motor function, reduced TNF‐α, IL‐6, oxidative stress, α‐synuclein aggregation, and restored dopamine levels in a rotenone‐induced Parkinson's disease rat model [126]. Albiglutide, dulaglutide, and DA5‐CH rapidly cross the BBB, while tirzepatide crosses slowly over several hours. This dual transport mechanism suggests that most incretin receptor agonists can quickly reach the brain and would be effective for AD and PD treatment [127].

**Table 2:** Summary of key studies on dual and triple incretin receptor agonists in AD and PD models.

Model	Agonist	Key findings	Mechanism
APP/PS1 mice [128]	DA3‐CH, DA4‐JC, DA5‐CH	Reduced amyloid, improved autophagy, and memory	Decreased inflammation, elevated neurotrophic factors
PD and AD [63]	DA5‐CH	Improved motor function, brain penetration	Reduced inflammation and amyloid burden
AD model [129]	DA4‐JC	Reduced cytokines, increased growth factors	Mitochondrial restoration and synaptic repair
AD model [124]	DA4‐JC	Increased synaptic density, restored mitochondria	Akt and JNK signaling pathway involvement
PD model [126]	Tirzepatide	Reduced alpha‐synuclein and inflammation	Restored dopamine, reduced TNF‐alpha, and IL‐6
In vitro and PK studies [127]	DA5‐CH, tirzepatide, albiglutide	DA5‐CH showed faster brain delivery	Superior BBB permeability
APP/PS1 mice [130]	Triagonist	Reduced amyloid, inflammation, and oxidative stress	Increased neurogenesis and BDNF
3xTg‐AD mice [131]	Triagonist	Improved cognition via CREB	Synaptic preservation and CREB activation
PD rats [132]	HM15211	Prevented neuron loss, reduced inflammation	Targeted nigrostriatal protection
SH‐SY5Y cells [133]	Triagonist	Increased survival markers, reduced apoptosis	Elevated cAMP, Bcl‐2, BDNF; reduced BAX

## 10 Triple Glucagon‐Like peptide‐1, Glucose‐Dependent Insulinotropic Polypeptide, and Glucagon Receptors (Gcgr) Agonists for AD and PD Treatment

Multiple receptor pharmacological approaches, like a balanced GLP‐1R/GIPR/GcgR triagonist, have shown better results on hepatic steatosis, glycemic control, and weight loss than dual agonists and single GLP‐1 receptor agonists in preclinical metabolic disease models (Table 2) [134, 135]. These drugs decreased β‐amyloid neuroinflammation and oxidative stress in the cortex and hippocampus while increasing BDNF and antiapoptotic Bcl‐2 and decreasing the proapoptotic protein BAX [130, 133]. In neuroblastoma cells, the GLP‐1R/GIPR/GcgR triagonist generates noticeably more cAMP than single GLP‐1R agonists, improving memory and cognition by activating the CREB pathway in 3xTg‐AD mice's hippocampus neurons [131]. In APP/PS1 mice, it decreases β‐amyloid neuroinflammation and oxidative stress while increasing neurogenesis, synapse number and BDNF expression [130]. The triagonist HM15211 reduces inflammation, α‐synuclein accumulation, and neurodegeneration in chronic PD rats, protecting dopaminergic neurons [132]. While triple and dual agonists induce cAMP in a similar way, triple agonists demonstrate superior neuroprotection against glutamate excitotoxicity [124, 128, 129].

## 11 Challenges Facing Incretin‐Based Therapies

Gastrointestinal adverse effects such as nausea, vomiting, and diarrhea are common with all GLP‐1 receptor agonists and are typically mild to moderate in severity in patients with type 2 diabetes mellitus (T2DM) [136, 137]. Similarly, GLP‐1 gastrointestinal side effects are associated with increased weight loss in patients with AD and PD. One of the major challenges is the limited ability of incretin‐based drugs to effectively cross the blood‐brain barrier and reach their target in the brain, which can hinder their efficacy in treating neurodegenerative diseases [138, 139]. While GLP‐1 and GIP analogs hold promise in modulating early neurodegenerative processes, their efficacy likely depends on timely intervention before irreversible neuronal loss occurs. Furthermore, optimizing the dosing, administration, and dosage of incretin‐based therapies for brain diseases is crucial for maximizing treatment effects and drug benefits [116, 140, 141].

## 12 Concluding Remarks

Current therapies for Alzheimer's and Parkinson's only relieve symptoms and are limited in efficacy. Targeting insulin and incretin pathways has shown promise, with incretin‐based medicines such as GLP‐1R agonists providing neuroprotective effects by lowering inflammation and oxidative damage. Dual and triple agonists, which can cross the blood‐brain barrier more effectively, provide considerably more neuroprotection. While GLP‐1 and GIP analogs hold promise in modulating early neurodegenerative processes, their efficacy likely depends on timely intervention before permanent neuronal damage occurs.

## Author Contributions

**Ousman Mohammed:** writing – original draft, writing – review and editing. **Tsehayneh Kelemu:** writing – review and editing.

## Conflicts of Interest

The authors declare no conflicts of interest.

## Transparency Statement

The lead author, Ousman Mohammed, affirms that this manuscript is an honest, accurate, and transparent account of the study being reported; that no important aspects of the study have been omitted; and that any discrepancies from the study as planned (and, if relevant, registered) have been explained.

## Data Availability

No data sets were generated or analyzed during the current study.
